# Competitive Examination in China

**Published:** 1883-02

**Authors:** 


### Competitive Examination in China.

An exchange says: "The yearly provincial examinations were held this autumn in Hongchow, the streets of which city are described as having literally swarmed with students of every age, from fifteen to eighty, and of all conditions, from the poor bank drudge to the sleek millionaire. The examination 'hall' was an enclosure of some eight acres, containing 10,000 cells for the students, each cell being about three feet wide, five feet deep, and seven feet high, and furnished with two boards—one for seat and bed, the other for desk and table. The candidates went in on the 8th day of the 8th moon, and remained in 'till the 10th—two nights and one day. They then came out, and returned on the 11th, when they went through precisely the same ordeal, which was repeated on the 14th, and they finally left their cells on the 16th. It is no unusual thing, we are told, for a candidate to be found dead in his cell."

HAD the sudden death of the Emperor Napolean III, which took place an hour before the time appointed for the administration of chloroform for the purpose of an operation, occurred, as it might well have done, while he was under the influence of the anæsthetic, it could hardly have failed to be set down to the temporarily debilitating action of the vapor on the heart. There would have been nothing in the results of the *post mortem* examination to contradict such a theory, as the appearances noted lead to the conclusion that death resulted from a sudden failure of the heart's action. The Emperor had taken chloroform four times, and had always passed under its influence satisfactorily, and suffered no inconvenience from its inhalation. It speaks highly for chloroform, and for the care with which its administration was regulated, that this should have been so in a case where there was so large an amount of organic disease, and where the heart subsequently showed itself so prompt to fail.—*Medical Record, London.*

